# The impact of the COVID‐19 pandemic on skin cancer incidence and treatment in England, 2020

**DOI:** 10.1111/bjd.20409

**Published:** 2021-08-01

**Authors:** Z. C. Venables, S. Ahmed, T. Bleiker, J. Broggio, M. Kwiatkowska, N. J. Levell, G. W. M. Millington, L. Paley, E. Payne, C. Proby, S. Vernon, S. McPhail

**Affiliations:** Norfolk and Norwich University Hospital Norwich UK; National Cancer Analysis and Registration Service Public Health England London UK; British Association of Dermatologists London UK; British Association of Dermatologists London UK; University Hospital of Derby and Burton NHS Foundation Trust Derby UK; National Cancer Analysis and Registration Service Public Health England London UK; National Cancer Analysis and Registration Service Public Health England London UK; British Association of Dermatologists London UK; Norfolk and Norwich University Hospital Norwich UK; Norfolk and Norwich University Hospital Norwich UK; British Association of Dermatologists London UK; National Cancer Analysis and Registration Service Public Health England London UK; National Cancer Analysis and Registration Service Public Health England London UK; Ninewells Hospital & Medical School University of Dundee Dundee UK; National Cancer Analysis and Registration Service Public Health England London UK; National Cancer Analysis and Registration Service Public Health England London UK

Dear Editor, The first UK national COVID‐19 lockdown began on 23 March 2020. Immediately, almost all outpatient healthcare service requests temporarily focused exclusively on urgent referrals and 2‐week‐wait urgent cancer referrals, with restrictions due to staff sickness, redeployment and changing work environments. Additionally, patient anxiety regarding attending appointments and perceived overburdening of healthcare resources resulted in fewer presentations.^1^

Technological advancements have arisen from challenging circumstances. The National Cancer Registration and Analysis Service (NCRAS), England, has developed a Rapid Cancer Registration Dataset (RCRD). Due to automated data feeds, lag time from diagnosis to registration has been reduced from 18 to 4 months; however, data have not been quality assured to the same standards and completeness.^2^, ^3^ We identify how the pandemic has affected skin cancer.

The RCRD^2^, ^3^ provides estimates of cancer incidence using data from January 2018 to November 2020. The main resource is multidisciplinary team meeting datasets, which are not a reliable source for basal cell carcinomas (BCCs) and cutaneous squamous cell carcinomas (cSCCs), effectively excluding them. RCRD tumour resection procedures are identified as a definitive treatment, for example an excision but not a diagnostic biopsy.

A separate tool to identify BCC and cSCC pathology reports received by NCRAS before registration was developed for quality assurance; it was not developed to report incidence and so should be interpreted cautiously.^4^ Pathology reports duplicate tumours when there are diagnostic biopsies, re‐excisions, recurrences and supplementary reports. Furthermore, these data have not been quality assessed and therefore they are best interpreted as a representation of workload rather than incidence. The date of the report used is the date of sample collection preferentially.

Both methods are crude and not the gold standard of tumour registration. Therefore they are not representative of the true incidence but are rather an early, rapid estimate. All reported proportions represent a comparison of the same month or period of the previous year.

In May 2020, melanoma diagnoses reduced to 54%, increasing to 83% by November 2020. During the 8‐month period from April 2020 to November 2020, melanoma diagnoses reduced to 72%, with 2671 fewer melanomas diagnosed. Likewise, a reduction was seen in the diagnosis of all malignant cancers (excluding nonmelanoma skin cancer, NMSC) (74%), prostate cancer (64%), breast cancer (73%) and lung cancer (88%) over the same period (Figure 1a). The proportion of resection procedures in May 2020 fell to 69%, with an increase to 87% by November 2020 (Figure 1b). Cancer Waiting Times first treatments for melanoma similarly fell to 58% in May 2020 and rose to 91% by December 2020 (Figure 1c). Pathology reports for cSCCs and BCCs in April 2020 fell to 58% and 22%, respectively. By September 2020 counts increased to 95% for cSCCs and 72% for BCCs (Figure 1d).

**Figure 1:**
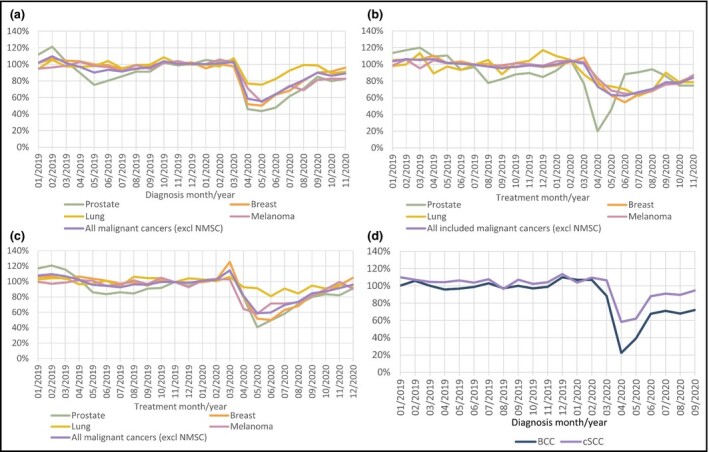
(a) Cancer diagnoses by cancer group and month, Rapid Cancer Registration Dataset (RCRD), England; working‐day‐adjusted. (b) Proportion of tumour resection procedures, by cancer group and month, RCRD, England. (c) First treatments by cancer group and month, Cancer Waiting Times data, England; working‐day‐adjusted. (d) Basal cell carcinoma (BCC) and cutaneous squamous cell carcinoma (cSCC) pathology reports received at the National Cancer Registration and Analysis Service, CancerStats2 keratinocyte cancer report, England. All data are comparisons with the same month in the previous year. NMSC, non‐melanoma skin cancer.

Undoubtedly, fewer cancer diagnoses are being made during the pandemic and an incomplete rebound is seen. Melanoma incidence decreased more than the incidence of all cancers overall (excluding NMSC); however, it is comparable with that of other cancers. BCCs are typically downgraded to routine pathways once diagnosed unless considered high risk, and therefore BCC pathology report counts drop substantially more than for cSCC.

Comparatively, as a proportion of activity for the same month of the previous year, in Australia, a reduction in surgical procedures to 82% for cSCC and BCC, and 75% for melanoma was seen at the pandemic onset, with a more rapid recovery.^5^ In Ontario, Canada, cSCC and BCC biopsies reduced to 82% and melanoma biopsies to 73% at the onset of the pandemic, with improvements seen in the following 10 weeks.^6^ In a study of 143 US practices, melanoma diagnoses fell to 30·4%, SCCs to 22·3% and BCCs to 14·1% in April 2020, but have since improved.^7^ Overall, a less substantial reduction in services is seen in countries where COVID‐19 counts remained lower.

The main limitation of these data is the lack of standard quality assurance as a result of attaining more rapid access to data. NCRAS report that the RCRD melanoma data reported in Figure 1(a) record 13% false negative (missing) and 9% false positive (additional) compared with formally registered cases in 2018·^2^, ^3^ Although crude, these data are essential to understanding the wider repercussions of the COVID‐19 pandemic beyond those directly infected. Early concerns precipitated the ‘help us, help you’ National Health Service campaign in October 2020,^8^ which promoted support for public access to healthcare services during the pandemic.

It is essential to ensure that skin cancer services continue, with virtual appointments playing an increasingly important role. It is of grave concern that the decline in cancer incidence represents patients who are likely to present later, resulting in worse morbidity and mortality outcomes. Further research must be undertaken to understand better the long‐term healthcare consequences of the pandemic to ensure we are able to prepare for further COVID‐19 waves and future national emergencies.

## Acknowledgments

the data for this study are based on patient‐level information collected by the National Health Service, as part of the care and support of patients with cancer. The data are collated, maintained and quality assured by the NCRAS, which is part of Public Health England. This article has been produced in partnership with the British Association of Dermatologists and Public Health England.

## Author Contribution

**Zoe Claire Venables:** Conceptualization (lead); Data curation (supporting); Formal analysis (supporting); Investigation (equal); Methodology (supporting); Writing‐original draft (lead); Writing‐review & editing (lead). **Shehnaz Ahmed:** Writing‐review & editing (equal). **Tanya O**  **Bleiker:** Supervision (equal); Writing‐review & editing (equal). **John**  **Broggio:** Supervision (equal); Writing‐review & editing (equal). **Marta**  **Kwiatkowska:** Writing‐review & editing (equal). **Nick J**  **Levell:** Supervision (equal); Writing‐review & editing (equal). **George W M Millington:** Writing‐review & editing (equal). **Lizz Paley:** Supervision (equal); Writing‐review & editing (equal). **Elsita Payne:** Data curation (equal); Formal analysis (equal). **Charlotte M**  **Proby:** Supervision (equal); Writing‐review & editing (equal). **Sally Vernon:** Supervision (equal); Writing‐review & editing (equal). **Sean**  **McPhail:** Formal analysis (lead); Methodology (lead).
